# Bacteriome of the Middle Ear in Children and Young Adults With Cholesteatoma and Retraction Pocket: A Pilot Study

**DOI:** 10.1002/oto2.70051

**Published:** 2025-02-13

**Authors:** Michal Bartos, Milan Urik, Lucie Buresova, Pavla Holochova, Eva Budinska, Petra Borilova Linhartova

**Affiliations:** ^1^ Department of Pediatric Otorhinolaryngology University Hospital Brno Brno Czech Republic; ^2^ Department of Pediatric Otorhinolaryngology Faculty of Medicine, Masaryk University Brno Czech Republic; ^3^ RECETOX‐Research Centre for Toxic Compounds in the Environment, Faculty of Science Masaryk University Brno Czech Republic

**Keywords:** bacteriome, children, cholesteatoma, chronic otitis media, middle ear, retraction pocket

## Abstract

**Objective:**

Chronic otitis media (COM) is a common middle ear disease in children and young adults. Dysfunction of the Eustachian tube and bacterial infection are the main causes. This pilot study aimed to describe and compare bacteriomes of the middle ear in children and young adults with serious forms of COM, such as cholesteatoma and retraction pocket (RP) of the tympanic membrane, with bacteriomes in healthy middle ears.

**Study Design:**

Observational study.

**Setting:**

Clinical practice in a tertiary center. From January 1, 2021 to August 31, 2022. Patients aged 0 to 20 years.

**Methods:**

In this case‐control study, middle ears were swabbed during surgery on children with cholesteatoma (N = 23) or RP (N = 26) and on children indicated for cochlear implant (N = 15, controls). Genomic DNA extraction was followed by creation of a 16S ribosomal DNA gene library and sequencing on a MiSeq instrument. Samples with relative abundance of at least one bacterial genus >20% were considered positive for the specific genus.

**Results:**

Bacterial diversity was generally low in the middle ear samples from patients with COM, with DNA content from 1 or 2 bacteria usually dominating in the sample. A significant difference in positivity for one or more bacterial genera was observed between patients with cholesteatoma or RP (38.8%) versus patients indicated for cochlear implants (6.7%).

**Conclusion:**

While middle ear bacteriomes in cases of cholesteatoma and RP differed from those of controls, findings in the 2 pathological conditions were similar. These results support the statement that the RP could be a precholesteatoma stage.

## Graphical abstract

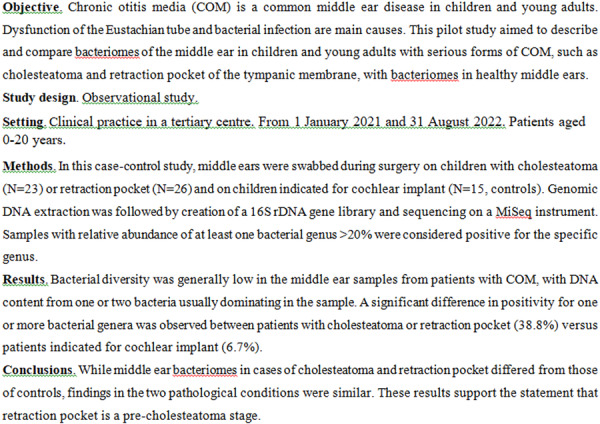

Chronic otitis media (COM) is a long‐term infection of the middle ear and one of the most common inflammatory diseases in otorhinolaryngology, especially in children. Eustachian tube dysfunction and chronic inflammation play key roles in the development of the retraction pocket (RP) of the tympanic membrane and the growth of cholesteatoma.^1^, ^2^ These conditions can lead to hearing loss and life‐threatening complications.^3^, ^4^

RP is characterized by the invagination of the atelectatic tympanic membrane into the spaces or structures of the middle ear.^5^ When RP progresses, irreversible changes may occur, such as RP's fixation to bony middle ear structures. In the case of fixed RP, there is a risk of vascular oxygenation loss on the mucosal cover of the ossicles. Erosion of the ossicle chain may occur. This can lead to conductive hearing loss. The most dangerous process is the accumulation of epidermis in the RP with loss of the RP's self‐cleaning function. This process is the basis of a precholesteatoma stage.^6^, ^7^ Increasing cholesteatoma formation creates potential danger for life‐threatening complications due to the ingrowth of cholesteatoma tissue through the bone into the intracranial space. The most serious complications of cholesteatoma, among others, include meningitis, epidural abscess, and brain abscess, especially in children.^4^ Patients with severe forms of COM often must undergo middle ear surgery.^8^

In routine clinical practice, microorganisms colonizing the middle ear are determined by conventional culture techniques followed by analysis using, for example, matrix‐assisted laser desorption/ionization‐time‐of‐flight mass spectrometry and/or quantitative polymerase chain reaction (PCR).^9^, ^10^ The bacteria most commonly associated with COM are *Pseudomonas aeruginosa, Staphylococcus aureus, Acinetobacter* spp., and *Corynebacterium* spp.^11^, ^12^ The DNA of nonculturable, poorly described, and rarely isolated microorganisms can be detected by the use of sequencing technologies, in particular 16S ribosomal RNA (rRNA) sequencing or whole‐metagenomic sequencing.^13^ These approaches help in studying the DNA composition of the sample bacterial community, the so‐called bacteriome.

Although several studies have focused on the COM bacteriome, no comparison has been published to date of the middle ear bacteriomes in patients with cholesteatoma and RP.^11^, ^12^ Based on the pathophysiological assumption that adhesive otitis with RP is a precursor to cholesteatoma, as well as the possible key role of middle ear dysbiosis in the development of all forms of COM, we hypothesized that middle ear bacteriomes of the 2 severe forms of COM are similar.^4^

To date, there has been ongoing discussion over whether the healthy middle ear is even colonized by bacteria.^14^ Westerberg et al had reported identifying no microorganisms in any healthy middle ear samples, either through standard culture or PCR testing.^14^ This was supported by the findings of Jervis‐Bardy et al.^15^ On the other hand, some studies using metagenomic sequencing techniques have reported healthy middle ear bacteriomes in both children and adults.^16^ Unfortunately, a lack of negative controls and internal standards makes it impossible to determine contaminants in a sample, these being most often associated with the external auditory canal (EAC) or nasopharyngeal areas.^15^ It is generally accepted that using negative controls and incorporating internal standards (informally known as “spike‐ins”) into DNA pools can mitigate the problems posed by contaminants and the use of relative abundance data to allow for approximating absolute abundances.^17^ In such studies, samples from “healthy” middle ears (without infection and inflammation) were obtained from patients undergoing ear surgeries for the likes of cochlear implant, stapes surgery, and translabyrinthine resection of a vestibular schwannoma.^15^, ^16^, ^18^

In our pilot study, we aimed to (i) describe and compare bacteriome profiles for 2 COM conditions (cholesteatoma and adhesive otitis media with the RP) using 16S rRNA amplicon sequencing, and (ii) compare the bacterial DNA content from middle ears of children and young adults with COM with that from healthy middle ears of children indicated for cochlear implant, the latter constituting a control group.

## Materials and Methods

### Study Design and Clinical Examination

This pilot study was designed as an observational study. Approval for the study was granted by the Committee for Ethics (06‐150120/EK, on January 15, 2020). Written informed consent was obtained from all study participants or their legal guardians in accordance with the Declaration of Helsinki.

Subjects were recruited from our clinical practice in a tertiary center. They consisted of all patients undergoing surgery for acquired cholesteatoma, RP of the tympanic membrane, stage II and III of retraction according to the Charachone classification (R. Charachone, *Rev Laryngol Otol Rhinol*, 1988), or cochlear implant between January 1, 2021 and August 31, 2022 at the direction of a single otologist experienced in middle ear surgery in children.

Excluded were patients with congenital or residual/recurrent cholesteatoma, as well as patients with antimicrobial treatment (either systemic or ototopical) within 6 weeks of surgery. General exclusion criteria were age more than 20 years and finding of cleft palate.

### Sample Collection

During the surgery, in COM patients swab biopsies were collected from the middle ear using sterile FLOQSwabs 501cs01 swabs (Copan Diagnostics) while not making contact with the EAC. In cochlear implant‐indicated patients, swab biopsies were taken after mastoidectomy and before electrode insertion from approaches into the middle ear cavity, facial recess, or *aditus ad antrum*. To ensure accuracy and consistency, all the sampling was conducted solely by the surgeon. The collected samples were promptly transported to the research laboratory and stored at −20°C until batch processing (Supplemental File S1, available online).

### Data Filtering, Diversity Calculation, and Statistical Analysis

To prepare for calculating diversity, we filtered out first amplicon sequencing variants (ASVs) that were unassigned at the phylum level, then ASVs of the mock community and ASVs of genera that did not occur in at least one sample with at least 10% relative abundance (after mock community removal). This filtration left us with a median of 42 ASVs (minimum‐maximum: 1‐178) per sample, including negative controls. Pearson's *χ* ^2^ and Fisher's exact test (for categorical variables) and Mann‐Whitney *U* test (for 2 groups in continuous variables) and Kruskal‐Wallis analysis of variance test with Dunn's post hoc test (for more than 2 groups in continuous variables) were used to compare demographic, clinical, as well as bacterial composition derived characteristics among the groups of interest. If the number of reads in a sample did not exceed the maximum number of reads of negative controls (28,319 reads per sample, counted after removing the spiked mock community from each sample) or if the relative abundance of any bacterial genera did not exceed 20%, then the sample was considered as “non‐positive for DNA from specific genus/genera” originating from the sample. Results were considered statistically significant at *P* < .05. All statistical analyses were performed using R Statistical Software (R version 4.0.5), with ComplexHeatmap (v. 2.13.1) package for heatmap plotting.

## Results

### Clinical Characteristics

**Figure 1:**
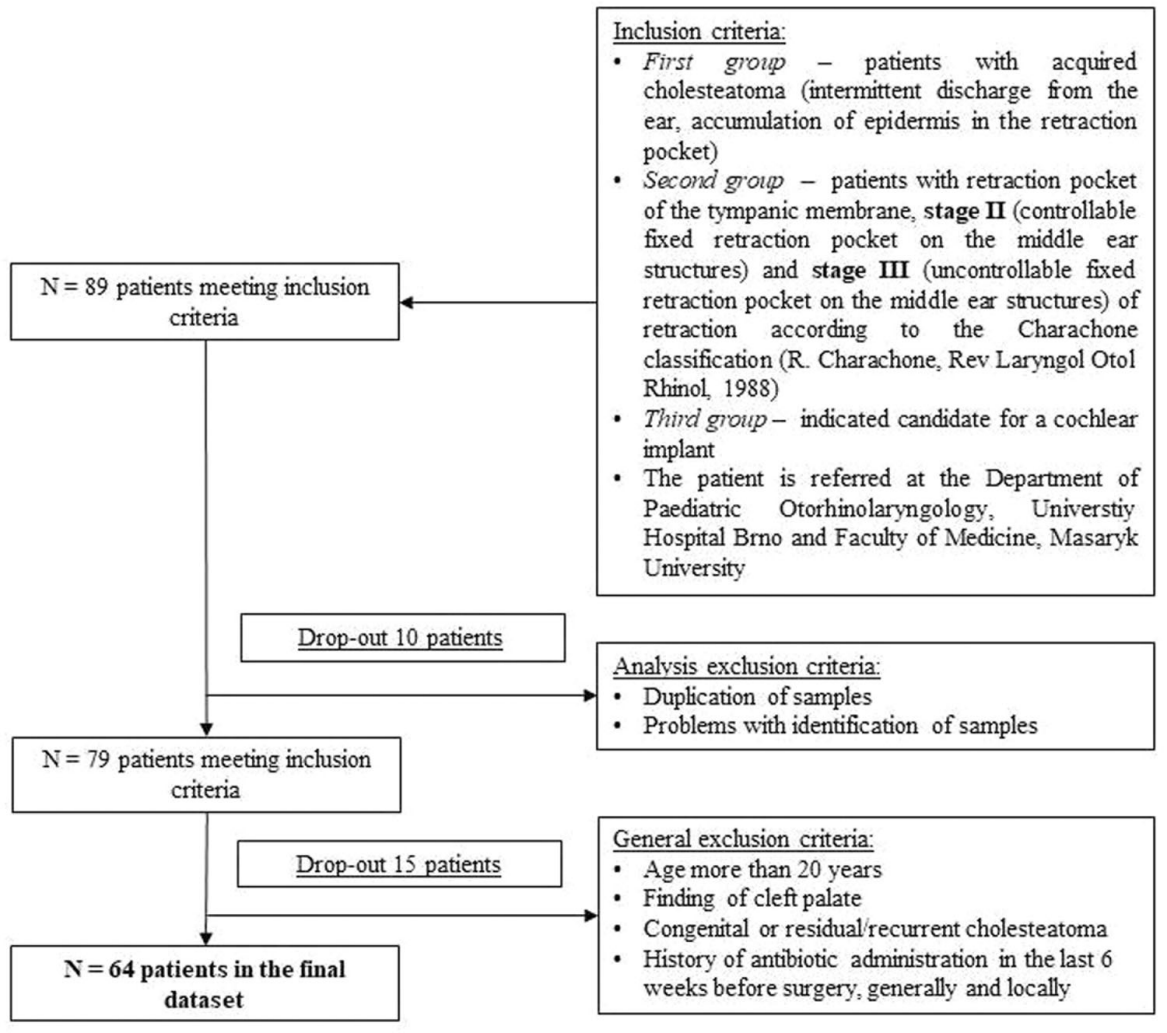
Flowchart of inclusion and exclusion criteria for the pilot study.

A total of 64 patients (41 male and 23 female) from the original 89 patients were included into this study after evaluation of the exclusion criteria (Figure 1). Regardless of sex, middle ear samples of diagnosed cholesteatoma were collected in 23 cases, samples of adhesive otitis media with RP on the tympanic membrane in 26 cases, and samples of patients indicated for cochlear implant in 15 cases. As shown in Table 1, the groups of patients differed significantly by age, adenoidectomy, and ventilation tube placement prior to enrollment in this study (*P* = .018, *P* = .031, and *P* < .001, respectively).

**Table 1:** Demographic and Clinical Characteristics of Studied Populations: Patients With Cholesteatoma, With Retraction Pocket, and Indicated for Cochlear Implant

Item	Cholesteatoma	Retraction pocket	Cochlear implant (controls)	*P* value	Statistical test
Number of patients in group (N)	23	26	15		
Median age, y (minimum‐maximum)	14 (2‐20)	13 (5‐18)	4 (0‐20)	.018	Kruskal‐Wallis ANOVA
Sex, male (N, %)	15 (65.2)	15 (57.7)	11 (73.3)	.597	Pearson's *χ* ^2^ test
Adenoidectomy (N, %)	15 (65.2)	17 (65.4)	4 (26.7)	.031	Pearson's *χ* ^2^
Ventilation tube (N, %)	15 (65.2)	23 (88.5)	4 (26.7)	<.001	Pearson's *χ* ^2^

Abbreviation: ANOVA, analysis of variance.

### Bacteriome Analysis

The minimum number of reads per spiked sample (including negative controls) was 8214. Relative abundances of *Allobacillus* and *Imtechella* from the mock community followed the expected ratio (1:1) in all samples. After removing the spiked mock community, the number of reads ranged between 3069 and 28,319 per sample in negative controls.

**Figure 2:**
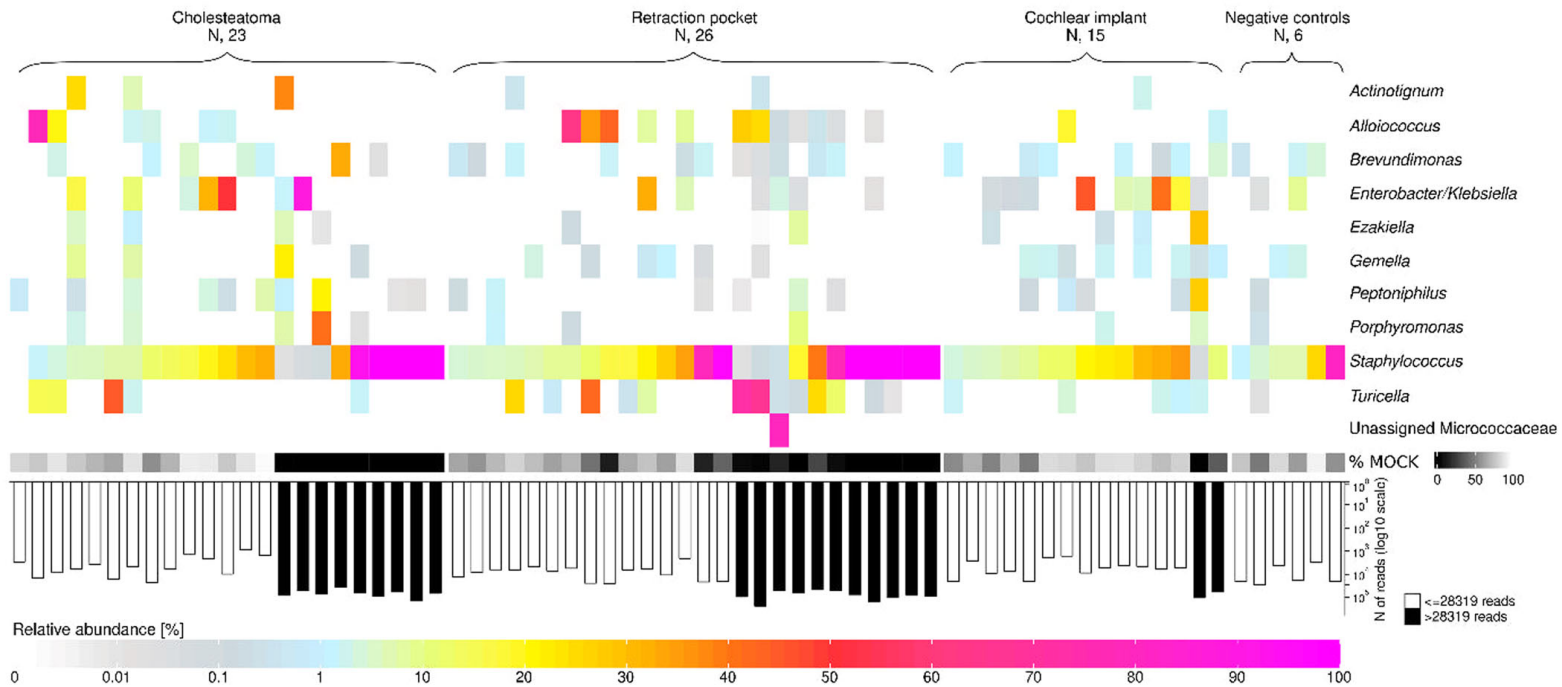
Heatmap of bacterial genera occurring in middle ear samples in relative abundance > 20% in at least 1 sample from patients with cholesteatoma, with retraction pocket, or indicated for cochlear implant. Negative controls are DNA‐free water. The sample was considered as “non‐positive for DNA from specific bacterial genus/genera” if the number of reads was <28,319 reads per sample (which was the highest number of reads per sample in negative controls) or if the relative abundance of any bacterial genus did not exceed 20%. This designation was applied after removing the spiked mock community from each sample. In addition, the relative abundance of the spiked mock community in each sample (prior to filtering) is shown. The low abundance of the mock community indicates enrichment of the bacterial DNA in the sample.

Of all the middle ear samples, 20 exceeded 28,319 reads and were positive for some bacterial genus (ie, the relative abundance of at least 1 bacterial genera exceeded 20%; Figure 2). With respect to proportion of patients positive for bacterial genus, the cholesteatoma and RP groups were similar (39.1% and 38.5%, respectively), as opposed to the group indicated for cochlear implant (6.7%) (*P* = .025, Fisher's exact test; Table 2).

**Table 2:** Categorization of Patients With Cholesteatoma or Retraction Pocket (Cases Grouped as Chronic Otitis Media, COM), and Patients Indicated for Cochlear Implant (Controls) According to the Finding of Bacterial DNA in Their Middle Ear Samples

Item	Cholesteatoma	Retraction pocket	COM (grouped cases)	Cochlear implant (controls)	*P* value
Number of patients in group (N)	23	26	49	15	
Nonpositive^a^ for DNA from specific bacterial genus/genera (N, %)	14 (60.9)	16 (61.5)	30 (61.2)	14 (93.3)	
Positive^a^ for some bacterial genera (N, %)	9 (39.1)	10 (38.5)	19 (38.8)	1 (6.7)	.063^b^ .025^c^

A negative control (DNA‐free water) went through the same process as did the patients' samples.

^a^ Sample was considered as “non‐positive for DNA from specific bacterial genus/genera” if the number of reads was <28,319 per sample (which is the highest number of reads per sample in negative controls) or if the relative abundance of any bacterial genera did not exceed 20%. This determination was made after removing the spiked mock community from each sample.

^b^ Comparison among groups of patients with cholesteatoma, retraction pocket and patients indicated for cochlear implant. Evaluated by Pearsons' *χ* ^2^ test.

^c^ Comparison between grouped cases (COM) versus controls (Cochlear implant). Evaluated by Fisher's exact test.

**Figure 3:**
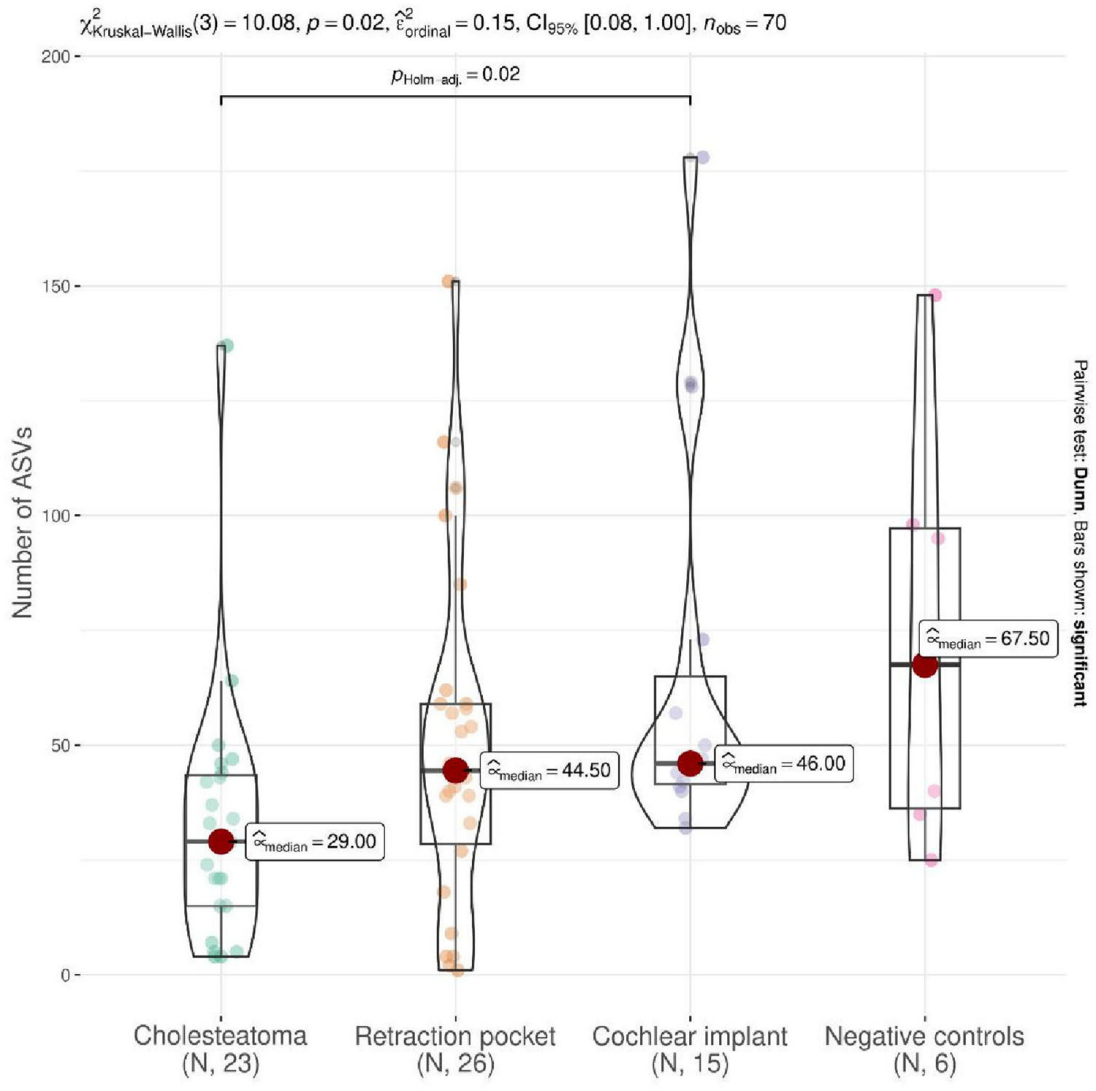
Comparison of alpha diversities between middle ear bacteriomes in patients with cholesteatoma, with retraction pocket, and indicated for cochlear implant. Also shown are negative controls (DNA‐free water). ASV, amplicon sequencing variant; CI, confidence interval.

Cholesteatoma patients had a lower median number of ASV, but this difference was significant only with respect to the cochlear implant group (*P* = .02, Dunn's test). In contrast, alpha diversities (Shannon index) of the middle ear bacteriomes were significantly lower in patients both with cholesteatoma and RP compared to those indicated for cochlear implant (*P* < .001, Dunn's test; Figure 3).

Eleven bacterial genera exceeded a relative abundance of 20% (Table 3). *Staphylococcus* was found in all negative controls (range of relative abundance 1.1%–80.6%) and *Brevundimonas* in 50% of negative controls (range 0.6%–3.6%; Figure 2). The relative abundance of *Staphylococcus* exceeded 20% in samples from 6 patients with cholesteatoma and 7 patients with RP (13/49 = 26.5%) compared to none in the group of patients indicated for cochlear implant (0/15 = 0%, *P* = .028, Fisher's exact test). Of all those samples with *Staphylococcus* in relative abundance >20% and comprising more than 28,319 reads, there was only 1 case (a patient with cholesteatoma) with co‐occurrence of another bacterial genus (*Brevundimonas* with relative abundance >20%) in the sample. Individual cases with relative abundance >20% of *Enterobacter*/*Klebsiella* in patients with cholesteatoma and Micrococcaceae unassigned in 1 patient with RP were identified.

**Table 3:** Bacterial Genera Found in Relative Abundance >20% in Middle Ear Samples From Patients With Cholesteatoma, With Retraction Pocket (Cases Grouped as Chronic Otitis Media, COM), or Indicated for Cochlear Implant (Controls)

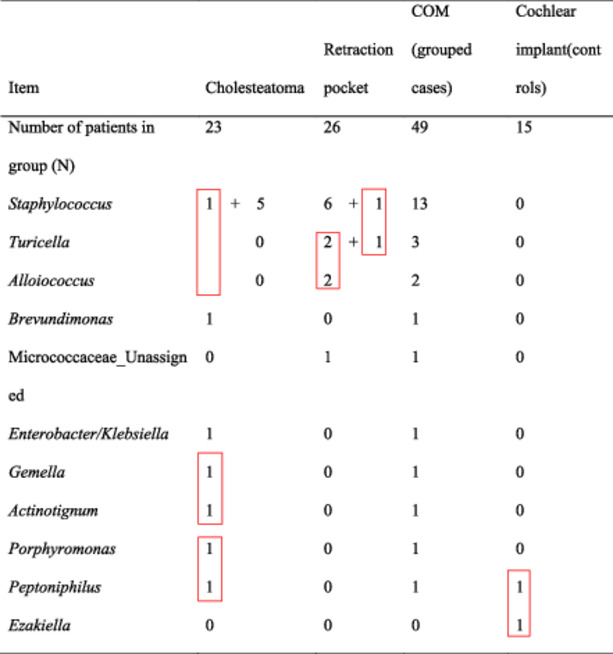

Red boxes indicate co‐occurrence of bacterial genera.

In 1 patient with cholesteatoma, a co‐occurrence of *Porphyromonas* and *Peptoniphilus* (both in relative abundance >20%) was recorded. Similarly, co‐occurrence of *Actinotignum* and *Gemella* was observed in 1 sample from a patient with cholesteatoma. *Alloiococcus* and *Turicella* co‐occurred in relative abundance >20% in 2 patients with RP. In only 1 sample from a group of patients indicated for cochlear implant, co‐occurrence of *Ezakiella* and *Peptoniphilus* (both in relative abundance >20%) was observed (Table 3).

## Discussion

COM is a multifactorial disorder that may be attributed to a combination of etiological factors, including microbial agents. Patient history of acute otitis media, allergy or atopy, an upper respiratory tract infection, snoring, and passive smoke also have been found to be risk factors for development of this disease.^19^ Using 16S rRNA gene‐based sequencing analysis, we found similar middle ear bacteriome profiles in both COM groups, the cholesteatoma and the adhesive otitis with RP. This result is in accordance with the assumption that RP could be a precholesteatoma stage, confirming our hypothesis.^6^, ^7^ Additionally, the bacteriome profile of the middle ear with COM differed from the bacteriome of the control group. The lower number of observed ASVs as well as alpha diversities of bacteriomes in the COM groups compared to the bacteriome profile of the middle ear in patients indicated for cochlear implants was expected inasmuch as an infection leads to a decrease in alpha diversity.^20^

Considering the risk of possible environmental contamination during DNA extraction and PCR amplification in the 16S rRNA sequencing, we strictly maintained sterile conditions and used negative controls as well as internal standards in our study.^21^ The findings in healthy middle ear samples of our children indicated for cochlear implants showed that the majority of samples appear without significant bacterial content, which is in agreement with the study published by Jervis‐Bardy et al.^15^ In line with Minami et al, our patients with COM without active inflammation (dry ear) had middle ear bacteriomes similar to those of patients with “healthy” middle ear.^21^

In middle ear samples from our patients with COM, the genus *Staphylococcus* was most often found in high relative abundance while in no case did it occur in middle ear samples from children indicated for cochlear implant. Our findings are in line with those of other studies, where *Staphylococcus* was associated with cholesteatoma.^22^, ^23^ This was to be expected inasmuch as the *Staphylococcus* genus may contribute to the middle ear pathology and can play a key role in the active wet middle ear inflammation with cholesteatoma.^21^ *S. aureus* in particular is one of the most abundant bacteria present in the chronic discharge due to bacterial superinfection of cholesteatoma.^6^, ^24^ Fujikawa et al had reported previously that *S. aureus* and *P. aeruginosa* are involved in cholesteatoma growth, but these bacteria were detected in the matrix of the cholesteatoma that was in contact with the EAC.^12^ It is known that *Staphylococcoci* are commensal bacteria of EAC and nasal mucosa.^21^, ^25^ Thus, it is important to avoid contamination from the EAC environment during sampling lest the subsequent analysis offer false positive findings and not reflect the true bacterial profile in the middle ear.^11^ The presence of *Alloiococcus otitidis* and *Turicella otitidis* also have been described in EAC.^26^ Xu et al reported microbiota of adenoid vegetation in children, such as *Haemophilus influenzae*, *Streptococcus pneumoniae*, and *Moraxella catarrhalis*.^27^ These bacteria are those most present in pathogenesis of acute otitis media and associated with recurrent otitis media.^28^ Enokson et al reported that microbial profile of otitis media with effusion (OME) in the pediatric population is dominated by *H. influenzae*, *S. aureus*, and *S. pneumoniae*, as well as *A. otitidis*.^26^ Ari et al evaluated microbiota of OME in children, finding that the most abundant bacteria were *A. otitidis*, *T. otitidis*, and *Staphylococcus auricularis*.^29^ It can be seen that the polymicrobial profile of OME is related to that of the nasopharynx via the Eustachian tube.^28^ Therefore, communication between the EAC and middle ear may influence the microbial profile of the middle ear. Nevertheless, the presence of *A. otitidis* and *T. otitidis* in the middle ear effusion, as otopathogens, may be involved in the anti‐inflammatory response in OME. In the nasopharynx, by contrast, these bacteria can trigger proinflammatory reaction.^26^ Neff et al found *A. otitidis* in samples of middle ear with cholesteatoma.^18^ Our finding of the *Alloiococcus* genus in the precholesteatoma samples, therefore, brings new insights into this disease's pathology. It may have some role in cholesteatoma development and should be closely observed in future studies.

We are also the first to report the presence of *Brevundimonas* genus in the middle ear. *Brevundimonas* spp. were previously described by Koeller et al in patients with diffuse chronic rhinosinusitis without nasal polyps,^30^ but a correlation between COM and chronic rhinosinusitis has not been described.

*Enterobacter* may play a role in COM pathogenesis. Albert et al reported the presence of this species in the chronic mastoid granulations when processing the granulations by standard microbial cultivation.^31^ Another study informed about patients with acquired cholesteatoma, where quinolones reduced the abundances of *Corynebacterium* and *Staphylococcus* genera, and diverse Proteobacteria (eg, *Haemophilus*, *Enterobacter*).^32^ These findings are in line with our results, where *Enterobacter/Klebsiella* was found in high abundance within the middle ear sample from 1 patient with cholesteatoma.

*Porphyromonas* was previously associated with the chronic suppurative otitis media, but this genus is less common in patients with COM. *Porphyromonas* is more common in other anaerobic infections, such as within the oral cavity, nasopharynx, or sinuses.^32^ In our study it was found only in a single patient with cholesteatoma. Liang et al, however, described *Porphyromonas bennonis* elevated in samples from patients with acquired cholesteatoma.^33^

Another genus found in patients with cholesteatoma was *Actinotignum* (family Actinomycetaceae). Könönen et al described the increased presence of Actinomycetes in nasopharynx of children suffering from recurrent otitis media during their first 2 years of life. In cases of COM and mastoiditis, *Actinomyces turicensis* was the most often detected.^34^

No probiotic or pathological effect of bacterial genera in recurrent otitis media has been observed.^35^ Some proinflammatory role of *Gemella* genus in the nasopharynx of children without acute otitis media was described by Xu et al, but no study has shown a relationship between *Gemella* genus and COM.^36^

Microbial profiles of healthy middle ear were described by Kalcioglu et al in 35 pediatric and 12 adult patients. The most abundant genera within samples collected from middle ear mucosa during cochlear implant surgery were *Propionibacterium* followed by *Streptococcus*, *Staphylococcus*, and *Ralstonia*.^16^ Moreover, middle ear samples from our patients indicated for cochlear implants showed us the presence of even more bacteria, such as *Ezakiella* and *Peptoniphilus*, but *Peptoniphilus* was present also in 1 patient with cholesteatoma. Minami et al report about pathogenic potential of the *Peptoniphilus* genus in the wet active inflammation of COM.^21^

This observational study provides the first report of middle ear bacteriomes with RP on tympanic membrane, adhesive otitis media, respectively. Possible limitations of our study include the age differences in the compared groups, especially between the control group and COM, and the fact that the sample size is not sufficiently large to ensure a better understanding and characterization of COM and healthy middle ear bacteriomes. Another limitation of this pilot study is the heterogeneity of our groups, especially the inclusion of children after adenoidectomy and with a history of ventilation tubes. This group with a history of ventilation tubes should be excluded from further study because of contamination of the middle ear space from the external ear canal. Contamination of the tympanic cavity through the Eustachian tube should be avoided, so the nasopharyngeal bacteriome needs to be known in the next study.

## Conclusions

Our results show similar bacteriome profiles in middle ears affected by severe forms of COM and support the claim that not only dysfunction of the Eustachian tube, but also persistent inflammation could be involve in their development, and that the RP of the tympanic membrane may be a potential precholesteatoma stage. Additionally, we found that the bacterial diversity is generally low in the middle ear samples of patients with COM, as these samples are usually dominated by 1 or 2 bacteria. Further study is needed with a focus on bacteriomes of the EAC, nasopharynx, and oral cavity while comparing these to the middle ear bacteriome of COM, such as in cases of cholesteatoma and adhesive otitis media with RP, and healthy middle ear. These findings could show us more precisely the role of bacteria in the pathogenesis of the growing cholesteatoma in the RP of the tympanic membrane.

## Author Contributions

**Michal Bartos**, clinical examination and sample collection, writing—original draft preparation, writing—original draft preparation; **Milan Urik**, conceptualization, clinical examination and sample collection, formal analysis, resources, writing—original draft preparation, supervision, project administration, funding acquisition; **Lucie Buresova**, data analysis, writing—review and editing, visualization; **Pavla Holochova**, sample processing, methodology, writing—review and editing; **Eva Budinska**, data analysis, data analysis supervision, writing—review and editing, visualization; **Petra Borilova Linhartova**, conceptualization, methodology, formal analysis, resources, data curation, writing—original draft preparation, visualization, supervision, project administration. All authors have read and agreed to the published version of the manuscript.

## Disclosures

### Competing interests

We confirm that this work is original and has not been published elsewhere, nor is it currently under consideration for publication elsewhere. None of the listed authors have conflicts of interest.

### Funding source

This work was supported by the Ministry of Health, Czech Republic, Conceptual Development of Research Organization (FNBr, 65269705) and by Masaryk University Brno (MUNI/A/1365/2022, MUNI/LF‐SUp/1058/2022). This publication was supported by the European Union's Horizon 2020 Research and Innovation Programme under grant agreement No. 857560. Authors also thank the Research Infrastructure RECETOX RI (No. LM2023069), the project CETOCOEN EXCELLENCE (No. CZ.02.1.01/0.0/0.0/17_043/0009632), and CETOCOEN Plus (CZ.02.1.01/0.0/0.0/15_003/0000469) financed by the Ministry of Education, Youth and Sports for supportive background. M.U. was supported by the NCMG research infrastructure (LM2018132 funded by MEYS CR) for sequencing. Computational resources were provided by the e‐INFRA CZ project (ID:90254), supported by the Ministry of Education, Youth and Sports of the Czech Republic.

## Supporting information

Supplementary Information
