# Supplementary material for: Bacteriome of the Middle Ear in Children and Young Adults With Cholesteatoma and Retraction Pocket: A Pilot Study
Source: OTO Open. 2025 Feb 13;9(1):e70051. doi: 10.1002/oto2.70051 (PMC11822790; doi:10.1002/oto2.70051)
Supplement: Supplementary file 1 — Supplementary Information [file OTO2-9-e70051-s001.docx]

DNA extraction

DNA from samples as well as from mock community ZymoBIOMICS Spike-in Control I (Zymo Research, Irvine, CA, USA) were extracted manually using the QIAamp DNA Mini Kit (QIAGEN, Hilden, Germany) according to the manufacturer’s instructions. The elution volume was 30 μl. Six negative controls (containing DNA-free water) were processed identically.

The purity and concentration of extracted DNA were determined using a NanoDropND-1000 spectrophotometer (Thermo Fisher Scientific, Waltham, MA, USA) and the quality of DNA was assessed using gel electrophoresis. Extracted DNA was stored at −20°C.

16S rRNA amplicon sequencing

All samples as well as the six negative controls were spiked with the mock community 2000× dilution). This non-human mock community has a defined bacterial composition (*Allobacillus* and *Imtechella*, 1:1) and is used as an internal positive amplification control.

PCR amplification was performed using the primer pair consisting of Illumina overhang nucleotide sequences, an inner tag, and gene-specific sequences targeting the V3-V4 region of the *16S rDNA* gene. The Illumina overhang served for ligation of the Illumina index and adapter. Each inner tag (i.e. a unique sequence of 12 bp) was designed to differentiate samples into groups. DNA was amplified utilizing the polymerase Q5 HighFidelity 2× Master Mix (New England BioLabs, Ipswich, MA, USA). PCR reactions were carried out in a total volume of 30 μl with the following conditions: initial denaturation 98°C/30 s; 30 cycles of 98°C/10 s, 55°C/15 s, 72°C/30 s; then final extension at 72°C/2 min. Negative and positive controls were included for PCR amplification. The amplified PCR products were determined by gel electrophoresis and subsequently purified using Agencourt AMPure XP Beads (Beckman Coulter, Brea, CA, USA).

Samples with different inner tags were equimolarly pooled based on fluorometrically measured concentration using a Synergy HTX fluorometer (BioTek, Winooski, VT, USA) and high sensitivity Quant-iT™ dsDNA Assay Kit (Thermo Fisher Scientific). Pools were used as a template for a second PCR with Nextera XT indexes (Illumina, San Diego, CA, USA). Differently indexed samples were equimolarly pooled based on fluorometrically measured concentration as previously. The prepared library was checked on a 2200 TapeStation Instrument using an Agilent D1000 ScreenTape System Kit (both Agilent Technologies, Santa Clara, CA, USA). The final library was diluted to a concentration of 4 nM and 20% of PhiX DNA (Illumina) was added. Sequencing was performed with the Miseq reagent kit V3 (600 cycles) using a MiSeq instrument according to the manufacturer’s instructions (Illumina).

DNA sequence analysis

Paired reads from 16S rRNA sequencing were first processed using an in-house pipeline implemented in Python 3 (python.org). Processing steps included trimming of low-quality 3′ ends of reads, removal of read pairs containing unspecified base N, and removal of pairs containing very short reads. In order to minimize sequencing and PCR-derived error, forward and reverse reads were denoised using the DADA2 amplicon denoising package in R ^19^. After denoising, the forward and reverse reads were joined into a single longer read using the fastq-join read joining utility ^20^. To be joined, reads in pairs had to have overlap of at least 20 base pairs and no mismatches were allowed. Pairs in which this was not the case were discarded. As the final step, chimeric sequences were removed from the joined reads using the remove Bimera function of the DADA2 R package. Subsequent taxonomic assignment was conducted by the uclust-consensus method from the QIIME microbial analysis framework using the Silva v. 123 reference database ^21,22^. The sequencing data was uploaded into the Sequence Read Archive (NCBI) under accession number PRJNA788869.
